# The use of electronic collars for training domestic dogs: estimated prevalence, reasons and risk factors for use, and owner perceived success as compared to other training methods

**DOI:** 10.1186/1746-6148-8-93

**Published:** 2012-06-29

**Authors:** Emily J Blackwell, Christine Bolster, Gemma Richards, Bethany A Loftus, Rachel A Casey

**Affiliations:** 1 Department of Clinical Veterinary Science, University of Bristol, Langford, Bristol, BS40 5DU, UK

## Abstract

**Background:**

The use of electronic training devices for dog training is controversial. The aims of this study were to give an indication of the extent to which dog owners use these devices in England, identify factors associated with their use, and compare owner report of outcomes. A convenience sample of dog owners in England was used to identify numbers using electronic training devices and identify reasons for use. Factors associated with use of remote e-collars only were determined by comparing dogs trained using these devices with two control populations matched for reason of use (recall / chasing problems). Comparison groups were: those using other ‘negative reinforcement / positive punishment’ training techniques, and those using ‘positive reinforcement / negative punishment’ based methods. A multinominal logistic regression model was used to compare factors between categories of training method. Owner reported success for use was compared using chi-squared analysis.

**Results:**

For England only, 3.3% (n = 133) owners reported using remote activated e-collars, 1.4% (n = 54) reported use of bark activated e-collars, and 0.9% (n = 36) reported using electronic boundary fences. In comparison with the e-collar group, owners using reward based training methods for recall / chasing were 2.8 times more likely to be female and 2.7 times less likely to have attended agility training. Owners using other aversive methods for recall / chasing were 2.8 times more likely to have attended puppy classes than those using e-collars. However, the model only explained 10% variance between groups. A significantly higher proportion of owners in the reward group reported training success than those in the e-collar group.

**Conclusions:**

In conclusion, a fairly low proportion of owners select to use electronic training devices. For a population matched by reason for training method use, characteristics of dogs, including occurrence of undesired behaviours do not appear to distinguish between training methods. Rather, owner gender and attendance at training classes appear more important, although explaining a relatively small amount of variance between groups. More owners using reward based methods for recall / chasing report a successful outcome of training than those using e-collars.

## Background

**Figure 1:**
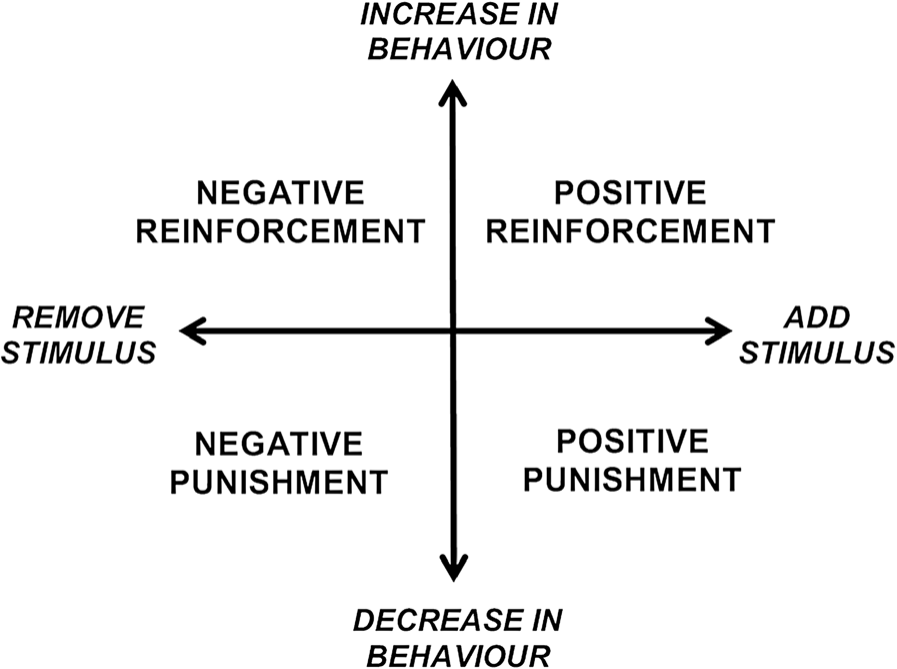
Diagram illustrating categories of reinforcement and punishment.

There are a wide range of training methods used in the training of dogs, and considerable debate about the relative benefits of using different approaches with respect to welfare implications [1], relationship with undesired behaviours [2] and efficacy [3]. Training methods can be broadly described with respect to definitions of reinforcement and punishment derived from psychological literature [4]. These are: positive punishment, where the probability of a behaviour occurring in the future is decreased when the behaviour is associated with application of a stimulus perceived as aversive; negative reinforcement, where the probability of a behaviour occurring in the future is increased when the behaviour is followed by the removal or avoidance of a stimulus perceived as aversive; positive reinforcement, where the probability of a behaviour occurring in the future is increased when the behaviour is associated with application of a stimulus perceived as rewarding; and negative punishment, where the probability of a behaviour occurring in the future is decreased when the behaviour is associated with the removal of a stimulus perceived as rewarding (Figure 1). In the authors’ experience, these terms often seem to be confused by dog owners, with the terms ‘reinforcement’ and ‘punishment’ perceived emotively rather than related to the increased or decreased likelihood of behavioural occurrence. In practice, positive punishment and negative reinforcement inevitably co-occur within the training environment, as do positive reinforcement and negative punishment, with the definition used dependent on the focal behaviour described. For example, in training a dog to walk to heel, pressure on a check chain positively punishes pulling behaviour, and release of pressure negatively reinforces walking to heel. Similarly, rewarding a dog with attention for sitting to greet people positively reinforces sitting, and withdrawal of attention if the dog does not sit would be negative punishment of the alternative behaviour. Due to the co-occurrence of these categories, in this study we have combined training techniques used by owners into ‘reward based’ (positive reinforcement and negative punishment, i.e. applying or removing stimuli perceived by dogs as rewarding) and ‘aversive based’ (positive punishment and negative reinforcement, i.e. applying or removing stimuli perceived by dogs as aversive).

Traditionally dog training relied heavily upon aversive based techniques, involving negative reinforcement or punishment. Although in more recent years increasing emphasis has been placed upon the use of positive reinforcement, aversive based techniques are still commonly used [3,5]. This includes those utilising an electric stimulus or pulse [6]. There are three types of electronic training device available to the general public [7]: those that are operated manually via a remote-controlled transmitter (hereafter ‘e-collar’); those that operate automatically in response to a dog barking (hereafter ‘bark e-collar’); and those that are activated at a boundary line to keep dogs within a defined area (hereafter ‘e-fence’). In all cases, the dog wears a collar with box containing the battery and circuits to provide a pulse of current between two electrodes on the ventral surface of the dog’s neck. The intensity and duration of the stimulus from e-collars can be varied and some collars, though not all, produce a warning beeping sound, prior to the shock. The shock lasts between 1/1000 second – 30 seconds and with a potential difference up to several thousand volts [8].

The use of electronic training devices is controversial. Currently, their use is banned in a number of European countries, including Wales [9], but not in other areas of the U.K. Those in favour of the use of such devices value their benefits for a number of reasons. In particular, they are suggested to be useful in correcting behaviour which is ‘self-rewarding’ such as chasing or hunting behaviour [10], as they cause a controlled sensation aversive enough to punish undesired behaviour which can be applied at a specific time contingent to the undesired behaviour and at a distance [7]. They are also suggested to facilitate the trainer teaching dogs and alternative behavioural response [7]. Further, advocates of electronic training claim that the use of these devices presents a smaller risk of long term welfare problems than alternative methods of punishment in general use.

Those opposed to their use suggest that e-collars cause unnecessary pain and suffering to dogs, through the application of an aversive stimulus [11]. It is also suggested that the poorly timed use of such devices by the general dog owner can cause anxiety in dogs [12], since unpredictable application of shock influences stress responses [13]. Dogs can also associate the application of the stimulus with events other than that intended [14], suggested to potentially result in the development of aggression [7,15], and reducing the desired effect of the stimulus. Anecdotally, there is also the potential for considerable abuse where owners activate the device in anger [16,17]. Furthermore, it is suggested that the use of e-collars are seen as an ‘easy fix’ for undesired behaviours, where a more considered approach with a deeper understanding of learning theory and dog behaviour would enable an ultimately more successful and welfare compatible resolution of undesired behaviour [8]. Many welfare [18,19], veterinary [15,17,20] and behaviour [21] organisations are opposed to the use of e-collars because of the welfare implications of their use, and the UK Kennel Club has campaigned against their use [16,22].

However, there is very little information available on the use of these devices in the UK. This study had three aims. The first was to estimate the number of owners using these types of devices in England. The second was to investigate whether there were particular owner and / or dog related factors associated with use of e-collars by dog owners, as compared to other training methods. The third was to indicate the relative success of different methods, as reported by owners.

## Methods

### **Questionnaires and subjects**

A standard questionnaire was developed to investigate the types of training technique used and the prevalence of undesired behaviours in a population of dog owners in the UK. Owners of multiple dogs were asked to only complete a single questionnaire, with respect to their youngest dog. The questionnaire, adapted from Blackwell et al. [2], was refined after piloting using a population of 15 dog owners in the Somerset area. The questionnaire was divided into four sections: owner demographics; dog demographics; information about training classes and training techniques used by owners with the focal dog; and information on the occurrence of a number of commonly reported undesirable behaviours in dogs.

In the first section data were collected using predominantly closed questions, and the options provided are shown in brackets after each variable: owner age (<25, 25–40, >40-60 and >60 years); owner gender (male, female), and experience in owing and training dogs (professional dog trainer; experienced dog owner with considerable training experience; experienced dog owner but limited experience in training dogs; inexperienced dog owner and trainer; not interested in dog training; other). This section also included an open question asking respondents to indicate which county in the UK they resided. The second section consisted of three closed questions and two open questions. Dog gender (male, female), neuter status (neutered, entire) and where the dog came from (breeder, re-homing centre, bred at home, or other for which respondents were given an open response section to specify) were closed questions. Breed and age of dog were asked as open questions. The third section included questions about the type of training classes attended. These were closed questions, and asked the respondent to indicate if they had attended puppy classes, obedience training classes, agility, flyball, gundog training classes, ringcraft classes or other types of training. Where these were selected, owners were also asked to complete two additional open questions: ‘How long did you attend?’ and ‘How old was your dog when you attended?’ In addition, owners were given a list of specific types of training technique. These were:

· Food rewards (giving a treat) when the dog does a correct behaviour

· Bark activated citronella collar (automatically spays strong smelling liquid to stop barking)

· Harness to prevent pulling on the lead

· Verbal punishment (e.g. telling off or shouting) when the dog does something wrong

· Shutting away (physically removing from the room, sometimes called “Time out”) when the dog behaves badly

· Stroking or patting when the dog behaves well, verbal praise

· Pet corrector (aerosol type spray directed at dog to interrupt unwanted behaviour)

· Electronic boundary fence to prevent the dog from wandering off the property

· Physical punishment (e.g. smacking) when the dog does something wrong

· Withhold treats or food when the dog does something wrong

· Ignoring (stopping giving the dog any attention when he or she does something wrong)

· Electronic training collar (to give a remove electronic correction when the dog does something wrong)

· Choke chain (metal collar that tightens on the dog’s neck) to prevent pulling on the lead

· Playing (e.g. throwing a toy when the dog does a correct behaviour)

· Physical Manipulation (e.g. pushing the bottom down) to encourage a correct behaviour

· Pulling back on lead when the dog pulls

· Bark activated electronic training collar (automatically gives electronic correction to stop barking)

· Water pistol (sprayed to interrupt a behaviour when dog does something wrong)

· “Husher” device that prevents the dog barking

· Clicker Training (using the ‘click’ sound, followed by a treat when the dog does a correct behaviour)

· Stopping forward movement or changing direction when the dog pulls on the lead

· Non- verbal sound distraction (e.g. a can of stones, ‘training discs’ or air horn) to stop the behaviour when the dog does something wrong

· Prong collar (metal chain with extensions that put pressure on dog’s neck when it pulls on the lead)

· Citronella Collar (to give a remotely initiated unpleasant smelling spray when the dog does something wrong)

· Other (please describe)

For each they were asked if the training technique was used, and where this was affirmative, additional questions were asked (‘Why did you decide to use this technique (e.g. used in training class, trainer recommended, found on internet)?; ‘For which behaviours did you use this technique (e.g. barking, pulling, not coming back when called)?’, and ‘Was the technique successful? (Yes, No)). In the final section, owners were given a list of 37 common undesired behaviours. These were not described as ‘undesirable’ but listed as brief descriptions to reduce the influence of subjective interpretation by owners as much as possible. For each behaviour, owners were asked to report whether the behaviour occurred currently (Yes / No), whether it had ever occurred in the past (Yes / No) and whether they considered the behaviour a problem (Yes / No). The behaviours requested are listed below:

· chew inappropriate items when you are present?

· chew or destroy anything when you are out?

· bark or howl when you leave the house?

· house soil when you are in the house?

· house soil whilst you are out of the house?

· bark or whine whilst you are in the house?

· growl at or bite other dogs within the household?

· bark, lunge, growl at or bite other dogs when out for a walk?

· hide or run away from family members?

· hide or run away from unfamiliar people?

· bark, growl at or bite family members?

· hide from or avoid other dogs when out of the house?

· bark, lunge, growl at or bite unfamiliar people in the house?

· bark, lunge, growl at or bite people you meet when out on walks?

· jump up to greet you?

· paw at you or demand attention in other ways?

· pull on the lead when on a walk?

· growl or bite when told off?

· wake you up in the middle of the night?

· eat faeces?

· chase things (e.g. cars, people, bikes)?

· eat excessively and vomit?

· always follow you around the house?

· steal food?

· steal objects?

· not come back to you when out for a walk?

· mouth hands, arms or clothing?

· show sexual behaviour towards people (e.g. mounting)?

· have a fearful response to noises (e.g. fireworks)?

· obsessively lick him/herself?

· keep spinning or whirling for no apparent reason?

· spin or whirl when told off?

· guard his/her food bowl?

· become very excitable with visitors?

· become very excitable when out?

· show excitable behaviours in many situations?

· become very excitable when told off?

A convenience sampling method was used to recruit dog owners to the study between May 2007 and August 2009. Dog owners out walking their dogs, attending agricultural shows and dog-related events, or visiting veterinary surgeries and pet shops in locations across UK were asked to complete the questionnaire (Table 1). Questionnaires were distributed with a reply paid envelope to maximise return rate. The protocol received approval as a study involving human participants from the local Ethical Review process at the University of Bristol.

**Table 1:** Distribution of owner questionnaires

**Type of questionnaire distribution**	**Number of questionnaires**	**Percentage of questionnaires**
Veterinary practices	835	21
Dog shows or related events	1941	50
Agricultural or horse shows	245	6
Dog walkers	539	14
Pet shops or other shops	239	6
Other or missing information	99	3

### **Statistical analysis**

Data were checked for coding and input errors, duplicates removed and implausible responses recoded as missing. The frequency and percentage of owners using electronic training methods were calculated. Most data were categorical. Age of dogs in months was not normally distributed but normalised by log10 transformation. Attendance at training classes was reduced to a 0/1 score, by including all dogs reported by owners as having attended the class for at least 4 weeks for all types of classes except for puppy classes, where attendance was scored only where owners reported attending for at least 2 weeks when their dogs were 12 weeks of age or less. The estimate of e-collar use was calculated as a percentage of respondents reporting use of bark activated, remote activated collars, and invisible boundary fence systems. Cases from Wales were excluded because these are no longer likely to be relevant given the recent ban of such devices. Cases from Scotland were also excluded because of small numbers of questionnaires distributed in this area.

In order to identify suitable cases and controls from the full dataset, the numbers of owners using e-collars for different reasons were identified. Electric boundary fence systems were not included due to low numbers reported, and their use in a specific context. Cases using remote and bark activated electric collars were combined for further analyses. Behaviours where remote devices were used in <20 cases were excluded, or combined into single variables were appropriate. Remaining categories where sufficient owners had used e-collars for analysis were recall problems /chasing behaviours combined, and barking. Comparison populations of owners who had specifically recorded using different training techniques for these behaviours were identified. To reduce the number of comparisons, individual training methods in comparison groups were combined into those which involved the application or removal of an aversive stimulus (positive punishment or negative reinforcement), and those which involved the application or removal of a rewarding stimulus (positive reinforcement or negative punishment). Comparison of multiple training methods was not considered appropriate for the questionnaire data since the relative extent to which training devices were used was not explored. It was considered more robust to use mutually exclusive groups in the use of training methods as it is more likely that the methods used were those predominantly chosen by the owner. Categories were made mutually exclusive by removing those cases where multiple techniques were used by owners for the same behaviour. As this left only a small number of cases where owners had exclusively used e-collars for barking problems (N = 14), further analyses were conducted only for different training approaches to recall / chasing behaviour. These cases all used remote activated e-collars.

A multinominal logistic regression analysis was used to investigate prediction of membership in each of the categories of training type (e-collar, other aversives, reward based), based on general factors such as owner and dog characteristics, attendance at training classes, occurrence of undesired behaviour (excluding recall / chasing behaviour) reported by owners, and the total number of behaviour problems shown by each dog calculated as a proportion of total number of behaviours possibly recorded on the questionnaire. Initially all potential risk factors were screened using univariable analyses, and only those showing significant difference between groups at P < 0.2 were included in the model. The contribution of individual components to the model was evaluated using the -2log likelihood. Individual variables were removed in a backward stepwise manner until the change in −2log likelihood in reduced models was less than expected for the associated degrees of freedom of the variable removed. The relative difference in risk as compared to reference categories was expressed as Odd’s Ratios.

Finally, a comparison was made of owner reported success of training using the training technique specified and the type of training used for recall training using cross tabulation and chi-square comparisons of groups.

## Results

### **Estimate of electronic training device use in England**

14,566 questionnaires were distributed direct to dog owners, of which 3897 (27%) were returned completed and legible. From those remaining, distributed in England, 3.3% (n = 133) owners reported using remote activate e-collars, 1.4% (n = 54) reported use of bark activated collars, and 0.9% (n = 36) reported using electronic boundary fences.

### **Factors associated with e-collar use**

From the entire population, 187 owners reported using either remote activated or bark activated e-collars. Of these, 185 had reported which behaviour they had specifically used the e-collar for. The types of behaviours trained with e-collars, and their reduction into categories for further analysis is shown in Table 2. Problems with recall / chasing and barking were identified as categories with sufficient number for further analysis. However, on removing cases where owners reported using more than one type of training technique, only 14 cases exclusively trained with e-collars remained for barking. Further analysis was therefore conducted for recall / chasing only. A comparison population of owners reporting the use of other training techniques for recall / chasing was identified as shown in Table 3. This resulted in a sample of 579 (83 using e-collar for recall / chasing, 123 using other aversives for recall / chasing, and 373 using rewards for recall / chasing) from which to investigate factors associated with e-collar use.

**Table 2:** Reasons cited by owners for remote e-collar and bark collar use

**Device**	**Reason for use**	**Number of owners using electric collar for this reason**	**Combined categories used in regression models**
E-collar (n = 185)	Chasing livestock	31	Problems with recall and chasing
	Chasing people (incl. bikes)	4	
	Chasing other dogs	5	
	Chasing cats	2	
	Chasing other (e.g. wildlife)	27	
	Recall	47	
	Barking	47	Barking, excluded as small numbers used exclusively
	General training	15	All categories excluded from regression analysis due to insufficient numbers
	Pulling on lead	9	
	Escaping / jumping at fence	3	
	‘Dominant behaviours’	1	
	Stealing food	1	
	Eating faeces	2	
	Aggression	2	
	Mounting other dogs	1	
	Jumping up	2	

Owners may have reported use for more than one situation.

**Table 3:** Categorisation of training methods used in comparison groups

**Training method used**	**Category**	**Recall /chasing problems**	
		**Number of cases**	**Number of cases exclusive to category**
E-collar	E-collar	102	83
Citronella collar			
Verbal punishment			
Water pistol			
Non verbal distraction			
	Aversive based	156	123
Withholding treats			
Ignoring			
Playing			
Clicker training			
Food rewards			
	Reward based	406	373

This table illustrates the specific training methods combined in the comparison groups, categorisation of training methods, numbers of cases where owners report the use of each category for recall / chasing behaviours, and the numbers included in further analysis which are mutually exclusive of other categories.

### **Description of population**

The general characteristics of the reduced sample of cases and controls where devices were used for training recall or chasing problems are shown in Table 4. Ages ranged from 2–190 months (mean 44). Overall 402 (69%) owners reported having attended some form of training class with the focal dog. 188 (33%) had attended puppy classes for at least 2 sessions when their dog was 12 weeks or less; 246 (43%) had attended obedience classes for at least 1 month / 4 occasions; 106 (18%) had attended agility classes for 1 month / 4 classes or more. Similarly, 14 (2%) attended flyball, 39 (7%) had attended gundog training classes and 98 (17%) had attended ring-craft classes.

**Table 4:** Description of general characteristics of the reduced sample of cases and controls where devices were used for training recall or chasing problems

**Variable**	**Categories**	**Number**	**%**
Distribution of questionnaire	Veterinary practices	137	24
	Dog shows or related events	265	46
	Agricultural or horse shows	34	6
	Dog walkers	95	16
	Pet shops or other shops	29	5
	Other or missing information	19	3
Gender of owner	Female	505	87
	Male	72	12
Age category of owners	Under 25 years	37	6
	25-40 years	135	23
	41-60 years	284	49
	Over 60 years	120	21
Owner location	Scotland or Wales	6	1
	North East	11	2
	North West	23	4
	East Midlands	49	9
	West Midlands	24	4
	East	62	11
	South East	80	14
	London	20	4
	South West	220	38
	Unknown	83	15
Owner report of experience	Professional dog trainer	24	4
	Experienced dog owner and trainer	202	35
	Experienced dog owner but new at training	248	43
	New or inexperienced dog owner	105	18
Origin of dog	Breeder	314	54
	Rescue centre	119	21
	Friend or relative	22	4
	Other (incl pet shops)	62	11
	Owner also breeder	61	11
Sex of dog	Males	286	49
	Females	292	50
Neuter status of dog	Neutered	286	49
	Entire	285	49
Breed type (split by UK Kennel Club categories)	Toy	21	4
	Terriers	60	10
	Utility	31	5
	Hounds	29	5
	Gundogs	184	32
	Working	39	7
	Pastoral	89	15
	Crossbreeds	124	21

No statistical difference (Kappa Measure of Agreement) was found with respect to distribution between categories in this sub-sample of owners using specific training methods for recall / chasing as compared to the whole population of owners surveyed (n = 3897).

### **Multinominal logistic regression model for risk factors for training method used by owners for recall / chasing**

Initial screening using univariable analysis resulted in the exclusion of: location type for questionnaire distribution, breed category of dog, cross breed or pure breed, age category of owner, origin of dog, attendance at gundog classes, attendance at ring-craft classes, age of dog, owner’s report of their level of dog owning and training experience, and all undesired behaviours except for house-soiling when the owner was out, waking the owner in the night and hiding from unfamiliar people.

Further reduction of the model was carried out in a backward stepwise manner. The final model was significantly able to distinguish between training categories (χ2 (8, N = 579) = 59.497, P < 0.001), with an overall correct classification rate of 64.1%. Evaluation of expected frequencies using cross-tabulations revealed no need to restrict model goodness-of-fit. The model fit was reasonable (χ2 (8, N = 579) = 9.481, P = 0.303) using a deviance criterion. Included in the final model with likelihood ratio tests were: gender of owner (χ2 (2) = 9.89, P = 0.007), attendance at puppy classes (χ2 (2) = 17.865, P < 0.001) and attendance at agility classes (χ2 (2) = 16.113, P < 0.001). The relative influence of these variables between comparison groups and the e-collar group is shown in Table 5. Values of R^2^ suggested that the variables in the model explained between 8.4% (Cox and Snell) and 10.1% (Nagelkerke) of the variance between categories.

**Table 5:** Multinominal logistic regression model of training category for recall / chasing behaviour

**Training group**			**Wald statistic**	**df**	**P value**	**Odds Ratio**	**95% CI for Odds Ratio**	
							**Lower**	**Upper**
Reward	Owner gender	Female compared to male reference	10.067	1	0.002	2.786	0.191	0.676
	Puppy class	Attending compared to not attending	0.156	1	0.693	1.124	0.498	1.589
	Agility class	Not attending compared to attending	10.101	1	0.001	2.711	1.466	5.014
Other aversive	Owner gender	Female compared to male reference	1.744	1	0.187	1.701	0.267	1.293
	Puppy class	Attending compared to not attending	9.916	1	0.002	2.817	0.186	0.676
	Agility class	Not attending compared to attending	0.136	1	0.713	1.134	0.580	2.220

Influence of individual variables included in the model on reward and aversive training method groups as compared to the e-collar trained group.

### **Owner reported success of training in different groups**

**Figure 2:**
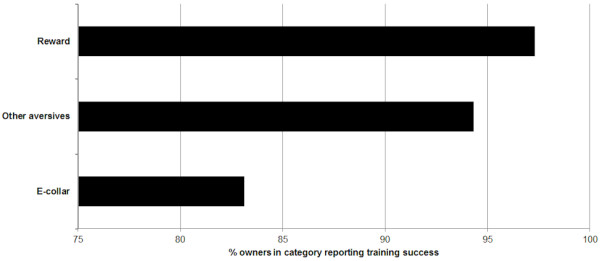
**Owner perceived success of training techniques.** Bar chart illustrating the proportion of owners perceiving their selected training method to be ‘successful’ for recall / chasing problems in their dog, split by category of training method.

Owner reported success of using the training method was significantly different between groups (χ2 (2, N = 579) = 26.999, P <0.001) with those training using e-collars reporting less success than expected, and those training with rewards reporting greater success than expected (Figure 2).

## Discussion

### **Estimated prevalence of electronic training device use**

The proportion of owners reporting use of electronic training aids is fairly low compared to other training methods, although extrapolation across the estimated UK dog population of 10 million [23] would suggest approximately 560,000 dogs trained with these devices. Should welfare implications arise from their use, therefore, the number of animals affected is considerable. However, some caution should be used in extrapolating these data across the UK. Data from Wales was removed due to the ban implemented in Wales [9] making these cases unlikely to remain relevant, and those from Scotland removed due to low numbers not supporting extrapolation. There were also some (non-significant at P < 0.2) differences between regions with respect to proportion of owners using e-collars. For example, a higher proportion in the East (7.3% cases) and North East of England (6.3% of cases) was found compared to London (1.7% cases), East Midlands (2.4% cases) and the South West (2.5%) cases. Since questionnaire distribution was not even across all regions it is possible that the overall estimate of prevalence may be affected by regional differences in use. Since a higher proportion of questionnaires were distributed in the South West where reported e-collar use was lower, regional effects may mean that the figure here is an under-estimate of overall UK use of devices.

Although questionnaires were distributed in as wide a range of environments as possible, this is not a random sample, and likely to have sampling biases which are difficult to quantify. For example, it is possible that the types of owners choosing to use electronic training devices may be more or less likely to be represented in the populations sampled, or may be more or less likely to complete and return questionnaires. These figures of e-collar use should therefore be regarded as an estimate, although they are analogous with figures published by the Electronic Collar Manufacturers Association who estimate 500,000 collar owners in the UK [24].

### **Risk factors for use of remote activated e-collars**

It is interesting that male owners were more likely than females to use e-collars compared to reward based methods for training their dog for recall or chasing problems. This may relate to gender differences in willingness to admit to e-collar use, or attitudinal differences to training techniques selected. Bennett and Rohlf [25] found that male owners were more likely to report that their dogs were ‘disobedient’ than females and so the increased use of e-collars reported by males in this study may reflect differences in attitudes towards potentially problematic behaviour. It is also possible that dogs show behavioural differences with owners of different genders [26].

In this study, reward based methods were more likely to be used by owners who had not attended agility classes. This may reflect preferences for training method use amongst proponents of this activity. However, further research is needed to investigate causality in this relationship, as it may reflect an attempt to resolve behavioural issues by increasing the dogs structured activities/mental stimulation/exercise by owners who also select to use e-collars.

Although there is no consensus in the literature regarding the influence of attendance at formal training classes on undesirable behaviour, a number of studies have suggested a reduction in problematic behaviour following attendance at obedience classes [27-30] and it seems inevitable that attendance at training classes, recommendations by trainers and observation of training methods used are likely to influence the subsequent selection of training methods by owners. It is therefore important that those running training classes have knowledge of the appropriate use of different training techniques and an understanding of the possible implications of their use.

Both Christiansen et al. [10] and Hansen et al. [31] suggest that different breeds of dogs differ in the extent to which chasing behaviour is motivated, efficacy of this type of training, and the level / number of applications of an aversive stimulus to modify behaviour. Here, breed type did not vary between groups, nor with owner reported success between groups. In the previous research relatively small numbers of animals were used, and of types unrepresented in this survey, making comparison difficult. The dogs selected for testing by Christiansen et al. [10] may not necessarily be representative of breed types reported here. Hence, whilst data here suggest that breed is not a strong factor in the choice of training method used for chasing or recall problems, further research is needed to investigate potential breed differences in response to different training methods.

Christiansen et al. ([10]) also suggest that the number of stimuli given to individual dogs related to a factor derived from tests considered to be related to ‘predatory motivation’. Specific behavioural characteristics such as predatory drive were not measured in this study, although owner reports of the number, occurrence of described undesired behaviours, and whether these were regarded as a ‘problem’ were measured and did not differ between groups.

No difference in age of dogs was found between groups in this study, although it has been reported that more coercive training techniques are used in older search and rescue dogs [32]. Differences between study findings may also reflect geographical differences.

### **Proportion of total variance explained by model**

It is salient that only between 8.4 and 10.1% of the variance between training methods used for recall or chasing problems is explained by the variables measured in this study. Hence, approximately 90% of the difference between categories is due to other factors, not measured here. It is possible that differences are explained by attitudinal factors in owners, their previous experience of different training methods, differences in relative ability to effectively apply and time interventions, and / or specific information or advice received from others, although further research is needed to investigate these factors further. In addition, the severity of the recall / chasing behaviour may have varied between groups. Although owners were asked if they considered the behaviour a ‘problem’, this is subjective, and not necessarily indicative of severity. Indeed, most owners with recall / chasing problems considered this to be ‘problematic’, presumably because behaviour of this type causes interruption of their daily routine, irritation or embarrassment.

### **Owner reported success of training techniques for recall / chasing problems**

A higher proportion of owners who had used reward based methods for recall / chasing problems reported success with their training. Although this may reflect increased efficacy when trainers use reward based methods, there are potential confounding effects in this comparison. For example, the relative training abilities of owners may differ with type of training method, there may be different perceptions of ‘success’ between different groups, or there may be differences in the initial severity of the problem for which different types of training method are selected which could affect outcomes. There is mixed evidence for relative efficacy from previous studies, although there is overall support for the conclusion that efficacy of electronic training devices is no greater than use of other methods.

In a population of owners attending a clinical behaviour service, owners reported the types of training techniques previously used and their perceived success [5]. A higher proportion of owners using reward based methods judged these to have had ‘positive effect’ and fewer ‘negative effect’ than those using more coercive methods. However, relatively few owners had used bark or remote activated e-collars. Eleven owners reported the use of such devices as having a positive effect, 6 a ‘negative effect’ and 6 ‘no effect’, although it is unclear for which behaviours these were used for, and the extent to which these behaviours were comparable to the use of other training techniques. In an observational study, Jones [33] investigated the use of e-collars to train dogs to stop attacking kiwis in a wildlife preservation programme. Thirteen dogs from a local pound underwent training, with the use of a stimulus as they approached a kiwi. However, on subsequent testing in a different context only one dog avoided approaching the kiwi.

Christiansen et al. [34] found a reduced likelihood of attacking sheep in a pen environment in which training with an e-collar had taken place the previous year (only 1 of 13 dogs which needed an intervention the previous year needed further training). However, no difference was found between dogs which had been given a stimulus and those which had not in a subsequent ‘path’ test, where dogs were presented unexpectedly with a lone sheep in a different context. Owners of all dogs in this study reported a reduced inclination to chase sheep, this was not influenced by whether they had received an electrical stimulus or not the previous year. According to owner report, only 1 of the 13 dogs given an electric stimulus a year previously had reduced or no interest in sheep, the rest reported as having no change. These findings may indicate that effects of e-collar training are not necessarily generalised: in other words the dog may respond as trained when in the specific context in which training has taken place, but retain the chasing behaviour in other situations.

In comparing the use of electronic bark collars with those using a citronella spray Juarbe Diaz and Houpt [35] reported that owners found the latter to have greater success at reducing unwanted barking. In addition, owner perception was that the e-collars were less humane to use on their dogs, although this may have had an impact on their evaluation of efficacy.

Christiansen et al. [10] found that dogs which had never seen sheep before had an increased chance of attacking sheep in a confrontation test compared to those which had experienced sheep – this may suggest that more dogs which chase sheep are those that are naïve to sheep rather than being established chasers. Apart from highlighting the importance of preventing such behaviour through careful introduction of puppies to livestock, dogs which chase through novelty / excitement may have their behaviour modified more easily than those with well-established chase responses. Nevertheless, apparently regardless of the extent to which the response is established, CABTSG [15] suggest that other training methods can be successfully used in those situations where e-collars are purported to be of greatest value (e.g. livestock chasing) and that successful resolution is regularly achieved by qualified individuals.

Some literature also compares the perception of owners more widely regarding the relative success of reward based and more coercive methods of training. For example, Loftus et al. [36] reported that across a range of undesired behaviours, owners reported reward based training as ‘more successful’ than other methods. Bussey [37] conducted an investigation of methods used in obedience training at a time when use of reward based training approaches were relatively new in this discipline. She suggested that dogs were no less successful where owners used reward based training rather than more traditional techniques, and that use of a fixed collar rather than choke / check chain had a positive influence on success.

## Conclusions

The results of this study suggest that a fairly low proportion of owners select to use electronic training devices. For a population matched by reason for training method use, characteristics of dogs including occurrence of undesired behaviours, do not appear to be important in distinguishing between training methods. Rather, owner gender and attendance at training classes appear more important, suggesting that owner attitudes and source of training advice may be the major determinants in choosing to use these types of training aid. More owners using reward based methods for recall / chasing report a successful outcome of training than those using e-collars.

## Authors' contributions

EB conceived the study, designed the questionnaire, collected data, conducted day to day supervision of the project, and contributed to drafting the manuscript. CB, GR and BAL were involved in questionnaire design and implementation, data collection, data entry and contributed to manuscript preparation. RAC participated in study design, conducted statistical analysis and drafted the manuscript. All authors read and approved the final manuscript.
